# Phanta: A Non-Fluorescent Photochromic Acceptor for pcFRET

**DOI:** 10.1371/journal.pone.0075835

**Published:** 2013-09-30

**Authors:** Craig Don Paul, Csaba Kiss, Daouda A. K. Traore, Lan Gong, Matthew C. J. Wilce, Rodney J. Devenish, Andrew Bradbury, Mark Prescott

**Affiliations:** 1 Department of Biochemistry and Molecular Biology, Monash University, Clayton Campus, Melbourne, Victoria, Australia; 2 Bioscience Division, Los Alamos National Laboratory, Los Alamos, New Mexico, United States of America; Russian Academy of Sciences, Institute for Biological Instrumentation, Russian Federation

## Abstract

We have developed an orange non-fluorescent photochromic protein (quantum yield, 0.003) we call Phanta that is useful as an acceptor in pcFRET applications. Phanta can be repeatedly inter-converted between the two absorbing states by alternate exposure to cyan and violet light. The absorption spectra of Phanta in one absorbing state shows excellent overlap with the emission spectra of a number of donor green fluorescent proteins including the commonly used EGFP. We show that the Phanta-EGFP FRET pair is suitable for monitoring the activation of caspase 3 in live cells using readily available instrumentation and a simple protocol that requires the acquisition of two donor emission images corresponding to Phanta in each of its photoswitched states. This the first report of a genetically encoded non-fluorescent acceptor for pcFRET.

## Introduction

GFP-like proteins are valuable tools used extensively in molecular cell biology research [1], [2]. These genetically encoded fluorescent probes undergo a series of post-translational modifications to form a chromophore shielded within a characteristic β-barrel. Isolation of new naturally occurring proteins in combination with protein engineering has resulted in the availability of a wide range of proteins having a variety of useful properties including the ability to alter their optical properties on exposure to light of specific wavelengths, a process called photoactivation [3]. One particular class of fluorescent proteins (FPs) is able to undergo reversible photoactivation. For example, Dronpa [4] undergoes reversible negative photoswitching and is converted from a bright green fluorescent ‘ON state’ to a non-fluorescent ‘OFF state’ on exposure to intense cyan light; exposure to violet light returns Dronpa to the fluorescent ON state. Other photoswitching FPs have different colour emissions such as the cyan mTFP.07 [5], red KFP [6] and red rsCherryRev [7]. The FPs rsCherry [7] and Padron [8] undergo positive photoswitching and are converted from a non-fluorescent to a fluorescent state with photoswitching light. Still other FPs such as IrisFP [9] and NijiFP [10] can switch between an OFF state and a green or red ON state. These proteins have numerous applications including tracking targets in live cells, use as probes for super-resolution microscopy [11] and photochromic Förster resonance energy transfer (pcFRET) [12].

Pairs of FPs (donor/acceptor) suitable for Förster resonance energy transfer (FRET) are the basis of many different biosensors useful for imaging cellular events in live cells [13]. FRET in such experiments is most often followed by dual-channel monitoring of donor and acceptor emissions when the donor/acceptor ratio is fixed. Other approaches are more complex and require monitoring of additional emission channels. A range of complementary FP pairs are available, some of which can be used together in the same experiment enabling multi-parameter imaging experiments [14]. The complexity of such multi-parameter experiments is limited by the number of different FPs whose emission can be separately detected. Although the availability of non-fluorescent genetically encoded acceptors such as REACh [15], [16] or Ultramarine [17] has the potential to increase the number of separate events that might be monitored in the same experiment, access to expensive instrumentation is required to determine fluorescence lifetimes and FRET.

pcFRET represents an alternative approach for measuring changes in FRET. In this approach, illumination of a photochromic acceptor is used to reversibly alter its absorbance spectrum, thereby changing the degree of spectral overlap with emission of the donor. Donor fluorescence is measured first in the presence of acceptor whose absorbance spectra has a large degree of overlap with donor emission, then in the presence of acceptor with a small degree of overlap with donor emission. This approach is commonly implemented using chemical dyes but was only recently demonstrated using the photoswitchable bright red FP, rsTagRFP [12].

eCGP123 is a bright green FP we engineered for extreme stability using a recursive evolutionary method that involved the sequential insertion of destabilizing loops into exposed portions of the protein followed by directed evolution to overcome the resulting fluorescence loss [18]. Stable proteins are generally more resistant to mutation, which is an advantage when attempting to evolve novel fluorescent properties in which the mutations causing new properties may also cause destabilization [19], [20]. In this paper we describe Phanta, a novel orange photochromic non-fluorescent protein, derived by mutation of eCGP123, which is particularly suitable for pcFRET. We demonstrate reversible pcFRET for a biosensor comprising Phanta and EGFP, and use it to monitor activation of caspase 3 in single live cells.

## Results

### Phanta is a non-fluorescent GFP-like protein

**Figure 1:**
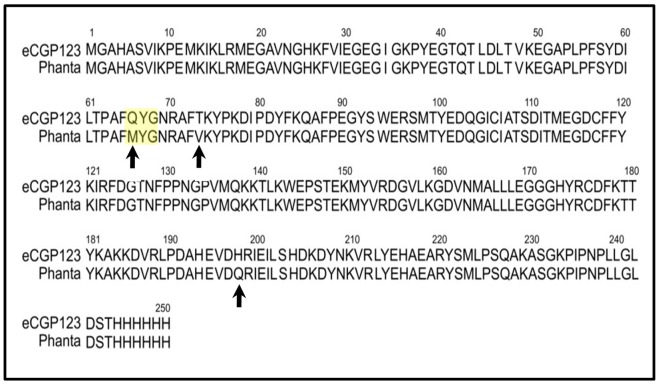
The amino acid sequence of Phanta. (A) The amino acid sequence for Phanta is shown aligned with its highly fluorescent parent eCGP123. The C-terminal His×6 tag used for protein purification is included in the sequence. Arrowheads indicate the three amino acid residues that differ between eCGP123 and Phanta. The chromophore tripeptide is highlighted by the yellow box. Figure generated by Kalign [31].

We sought to develop a non-fluorescent genetically encoded acceptor protein whose light absorbing properties could be usefully altered by exposure to light of specific wavelengths. eCGP123 is an extremely thermostable FP that without exposure to photoswitching light is bright green fluorescent (λ_max_^ex^, 495 nm; λ_max_^em^, 505 nm, fluorescence quantum yield (Φ_F_), 0.8) [18]. eCGP123 has reversible negative photoswitching properties and can be converted by exposure to cyan light from its normally bright green fluorescent ON state to a non-fluorescent OFF state. Exposure to violet light promotes return of eCGP123 to its ON state. During attempts to evolve eCGP123 to a more red-shifted protein, a bacterial colony expressing one particular variant appeared bright orange in colour under daylight conditions and black (i.e. non-fluorescent) when viewed under cyan and blue light illumination. This new variant was named Phanta due to its bright orange colour. Compared to the parent eCGP123, Phanta contained the three amino acid substitutions, Q66M, T73V and H197Q (Figure 1), each of which are predicted to be in close proximity to the chromophore in the X-ray crystal structure [21]. The substitution Q66M is in the chromophore tripeptide. The possibility that Phanta was also capable of photoswitching prompted us to investigate its properties further.

**Figure 2:**
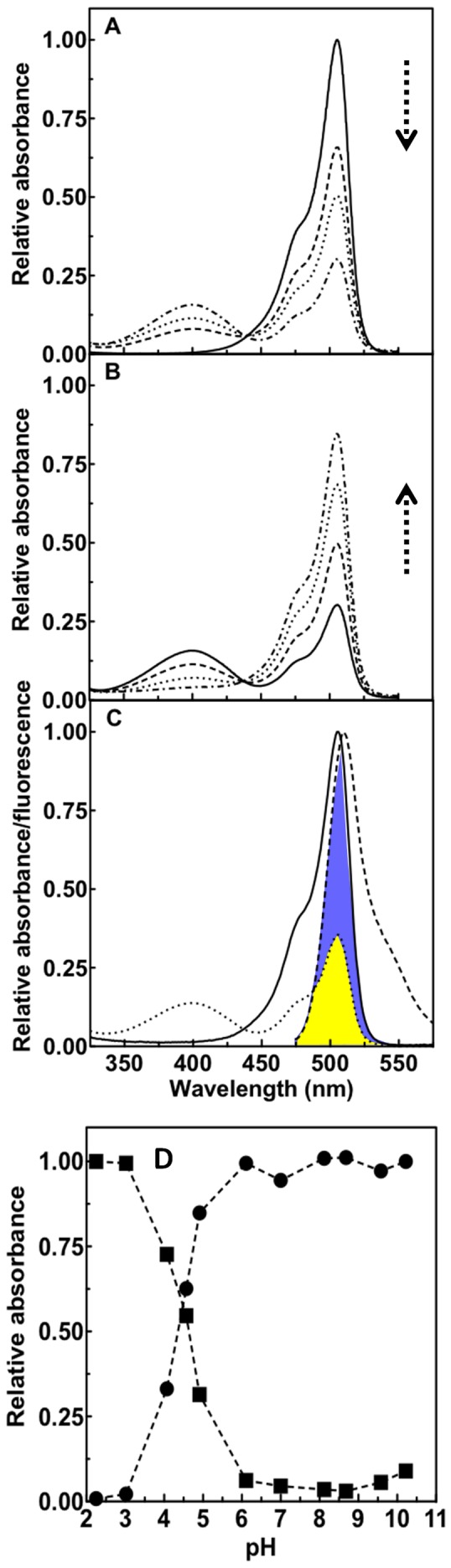
Some optical properties of Phanta. (A) The absorbance spectrum of Phanta initially in the ON state was determined before (solid line) and after (broken lines) different timed exposures to photoswitching cyan light from an LED (λ_peak_, 505 nm). The vertical arrow highlights the decrease in absorbance at 505 nm as a result of increased exposure to light. (B) The absorbance spectrum of Phanta initially in the OFF state was determined before (solid line) and after (different broken lines) different timed exposures to photoswitching violet light (λ_peak_, 405 nm). The vertical arrow highlights the incremental increase in absorbance at 505 nm as a result of increased exposure to light. (C) The absorbance spectra for the ON (solid line) and OFF (dotted line) states of Phanta are shown overlaid with the fluorescence emission spectrum (dashed line) for EGFP (donor). The overlap of Phanta absorbance in the ON (blue fill) and OFF (yellow fill) states with EGFP emission is shown. (D) Phanta in its ON state was titrated for pH and the absorbance determined at 505 nm (circles) and 390 nm (squares).

Phanta protein bearing a C-terminal His×6 tag was expressed in bacteria and purified, and the absorbance and fluorescence excitation/emission spectra determined. For Phanta not exposed to photoswitching light a major absorbing species (λ_max_^abs^, 505 nm; ε, 98,000 M^−1^.cm^−1^) was observed (Figure 2A) with a shoulder at ∼470 nm. Fluorescence emission (λ_max_^ex^, 505 nm;λ_max_^em^, 516 nm) was extremely weak (Φ_F_, 0.003). The optical properties of Phanta are summarized in Table 1, and compared to both eCGP123, the parent protein, and Dronpa, a well characterized brightly fluorescent reversible photoswitching protein [22]. These results show that compared to eCGP123 the Φ_F_ for Phanta is reduced by ∼260 fold whilst the ε is increased ∼1.6 fold and the λ_max_^abs^ red shifted by 11 nm.

**Table 1:** Optical characteristics of Phanta and eCGP123.

	λ_max_^Abs^ (nm)	λ_max_^Ex^ (nm)	λ_max_^Em^ (nm)	ε^λmax^ (M^−1^.cm^−1^)	Φ_F_	pK_a_
**Phanta**	506	506	516	98,000	0.003	4.5
**eCGP123**	495	495	505	59,900	0.8	6.0
**Dronpa** [22]	503	505	518	95,000	0.85	5.0

The effect of pH on Phanta absorbance was determined. Absorbance at 505 nm was stable over the range pH 5.5–10.5 but decreased at lower pH values giving rise to a 390 nm absorbing species (Figure 2D). A pK_a_ of 4.5 was calculated for Phanta which is significantly lower than that for the parent eCGP123. These results indicate that the absorbance of Phanta (λ_max_^abs^, 505 nm) and its use as a pcFRET acceptor will not be significantly compromised by pH values normally encountered in living cells.

### Phanta is photochromic

We next investigated the photoswitching properties of Phanta. The absorption spectrum for Phanta was determined before and after exposure to cyan light (λ_peak_,505 nm) produced by a high power LED. The 505 nm absorbing species decreased upon exposure to cyan whilst a new absorbing species (λ_max_^abs^, 390 nm) was observed to increase in amount (Figure 2A). Subsequent exposure of Phanta to violet light (λ_peak_, 405 nm) resulted in a reduction of the 390 nm species and a corresponding increase in the 505 nm species (Figure 2B). A single isosbestic point was observed at 435 nm. These changes in absorbance spectra for Phanta on exposure to photoswitching light are similar to those reported for Dronpa, a well-characterized negative photoswitching FP that reversibly photoswitches between a brightly fluorescent ON state (λ_max_^abs^, 503 nm; λ_max_^em^, 518 nm) and a non-fluorescent OFF state (λ_max_^abs^, 390 nm) on exposure to intense cyan light [4]. Although non-fluorescent we use here the same terminology to describe the different photoswitched states of Phanta, where the 505 nm absorbing species predominates in the ON state and the 390 nm absorbing species predominates in the OFF state.

**Figure 3:**
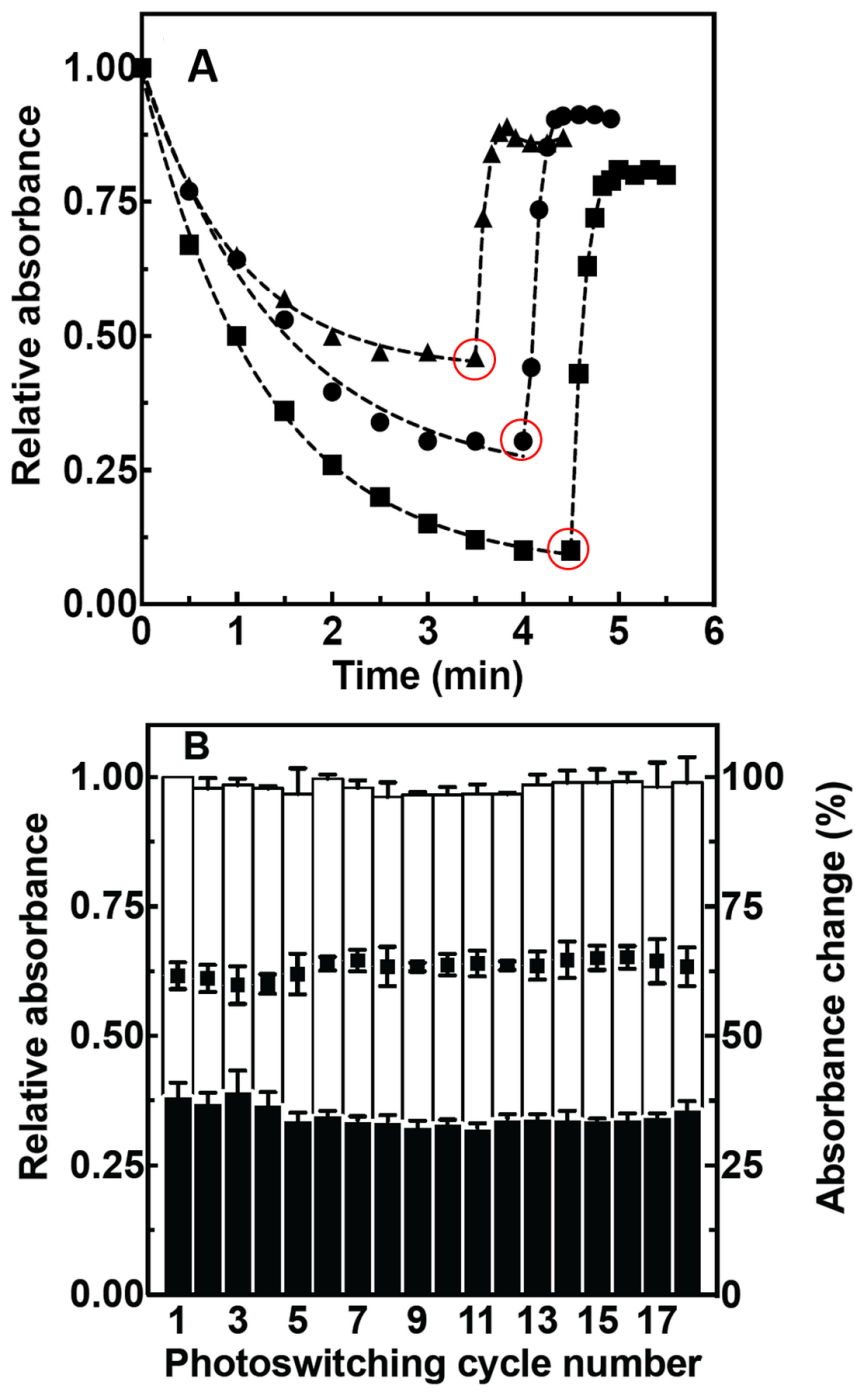
Some photoswitching properties of Phanta and EGFP^C3^Phanta. (A) The absorbance at 505 nm (λ_max_^abs^) for eCGP123 (triangle), Phanta (circle) and Dronpa (square) was determined for proteins in solution in the well of a microtitre plate after exposure first to photoswitching cyan light, then photoswitching violet light. The change from cyan to violet photoswitching illumination occurred after the symbol circled in red. (B) A solution of Phanta in the well of a microtitre plate was subjected to repeated cycles of photoswitching by exposure to defined periods of cyan or violet photoswitching light provided by an LED. Absorbance (505 nm; ON-state) is shown for each cycle after exposure to cyan light (filled bar) or violet light (open bar). The change in absorbance for each photoswitching cycle is shown (filled squares). Data plotted as the average of three separate experiments ± s.e.m. are shown.

We next investigated the rate at which Phanta underwent photoswitching. Absorbance corresponding to the ON state (λ_max_^abs^) for Phanta, the eCGP123 parent and Dronpa was followed and compared over a single cycle of photoswitching using LED illumination. The results show that, compared to eCGP123, Phanta photoswitched more readily on exposure to cyan illumination of this particular intensity, whereas Dronpa photoswitched more readily and to a greater degree when compared to both Phanta and eCGP123 (Figure 3A). Each of the proteins in the OFF state were rapidly converted to the ON state on exposure to violet photoswitching light.

### The effects of repetitive photoswitching on the stability of Phanta

A pcFRET acceptor should be capable of repeated cycles of photoswitching without appreciable loss in ability to absorb light. The absorbance of Phanta in solution was determined after alternate illumination with cyan and violet light. The results show that relative absorbance λ_max_^abs^, 506 nm) could be maintained over at least 18 cycles of photoswitching (Figure 3B). Collectively these results suggest that Phanta can undergo repeated cycles of photoswitching without significant loss of absorbance.

### A protocol for measuring FRET efficiency using Phanta and EGFP

We next investigated the suitability of Phanta as a pcFRET acceptor for use with green emitting FPs, such as EGFP, as it is particularly well suited (λ_max_^em^, 518 nm). Compared with the absorbance spectrum of Phanta in the ON state, the degree of overlap between the absorbance spectrum of Phanta in the OFF state and the EGFP emission spectrum is significantly reduced (Figure 2C). Förster distances of 52 Å (R_0_^on^) and 42 Å (R_0_^off^) were calculated for EGFP and Phanta in the ON state and OFF state, respectively. This suggests that for EGFP in close proximity to Phanta, significant alterations in donor emission will result on photoswitching of Phanta between the ON and OFF states. Such changes in donor emission on photoswitching can be used to estimate FRET efficiency.

**Figure 4:**
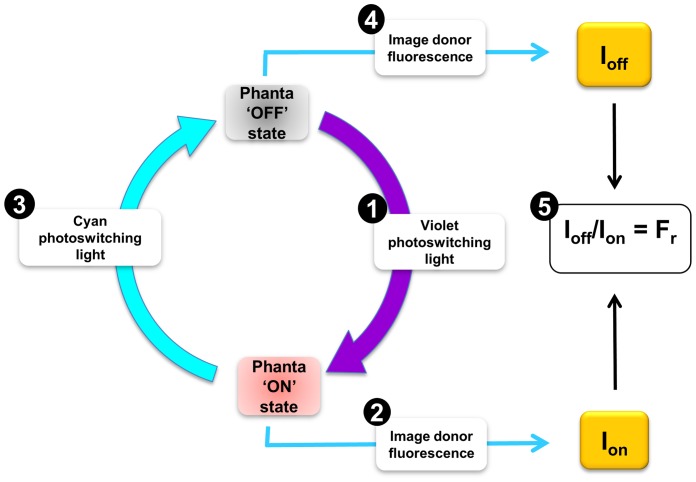
A scheme for monitoring FRET using Phanta and EGFP as a pcFRET pair. The steps (numbered) used in this study to estimate pcFRET efficiency (F_r_) in cells expressing EGFP^C3^Phanta are shown. (1) The sample is first exposed to photoswitching violet light (395–425 nm) to ensure the acceptor is fully in the ON state; (2) A donor fluorescence emission image (I_on_; 505–560 nm) is acquired using the readout laser (488 nm); (3) The sample is exposed to photoswitching cyan light (460–490 nm) to drive the Phanta into the OFF state; (4) Donor fluorescence emission image (I_off_) is acquired; (5) Corrected donor fluorescence images I_off_ and I_on_ are used to calculate F_r_ (I_off_/I_on_), an estimate of pcFRET efficiency. The steps 1–5 are repeated at desired time points.

A simple robust fluorescence microscopy protocol was developed to determine pcFRET in live cells expressing a Phanta/fluorescent donor combination (Figure 4). Samples were first illuminated with photoswitching violet light to ensure Phanta was fully in the ON state, and therefore could act as acceptor of green emissions. Donor fluorescence emission was imaged (I_on_) with cyan excitation light using an intensity unable to switch Phanta into the OFF state. Phanta was then intentionally driven into its OFF state using by illumination with photoswitching cyan light, and then donor fluorescence imaged once more (I_off_) under the same conditions used to acquire I_on_. A ratio of the two background-corrected images (I_off_/I_on_) was used as a measure of pcFRET efficiency (F_r_). This sequence of events was repeated for each new determination of FRET. Typically, one complete cycle to determine F_r_, when performed with manual filter changes, required ∼10 s.

### EGFP^C3^Phanta as a FRET biosensor for caspase 3 activity

**Figure 5:**
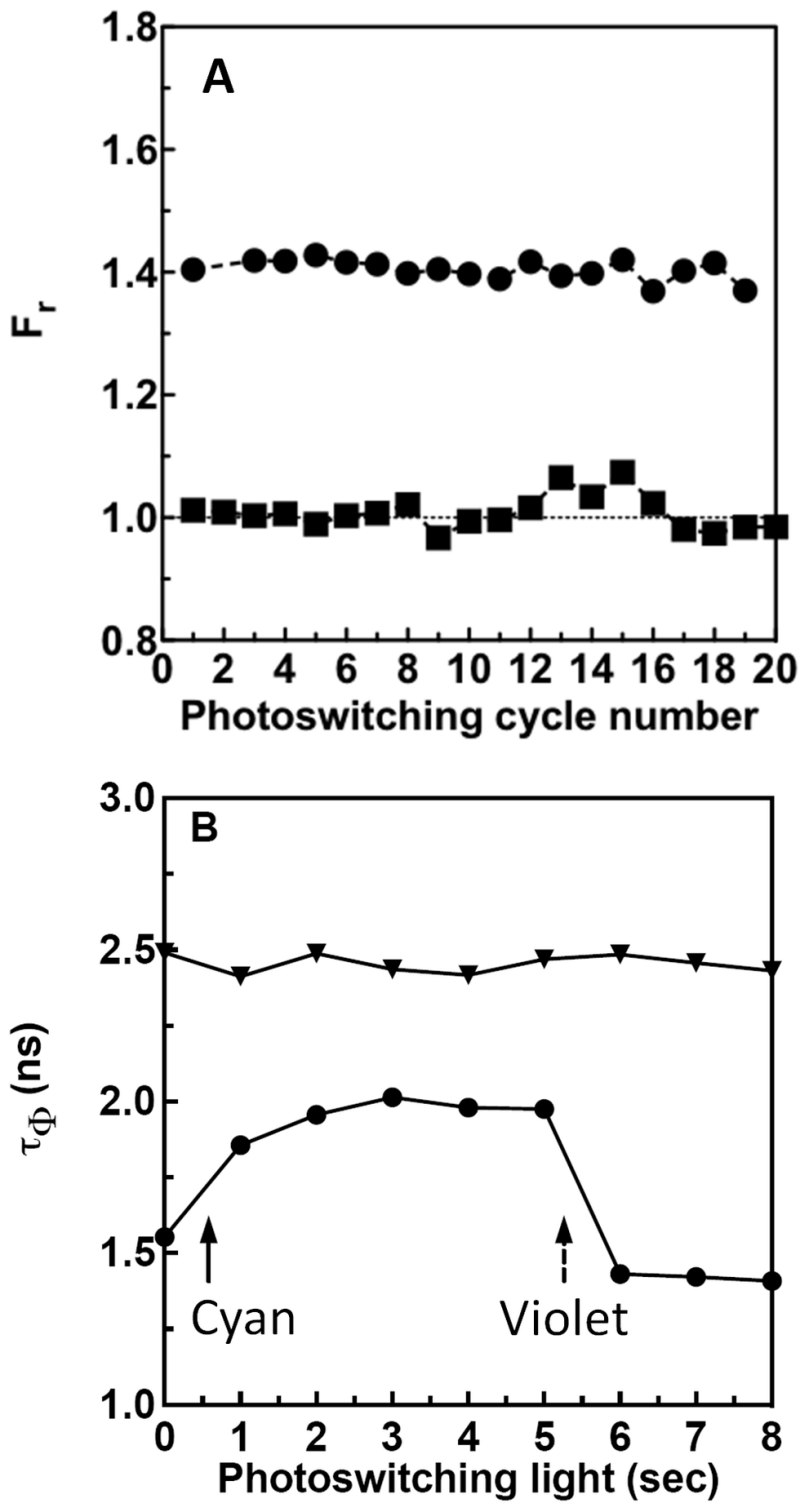
Monitoring FRET for EGFP^C3^Phanta. (A) EGFP^C3^Phanta or EGFP bound to Ni-NTA beads was repeatedly photoswitched (20 complete cycles) by exposure to defined periods of cyan or violet photoswitching light from a fluorescence microscope. Using the protocol summarised in Figure 4 fluorescence images (I_on_ and I_off_) of an individual bead were acquired and used to calculate an average F_r_ values for EGFP^C3^Phanta (circles) or EGFP (square). (B) EGFP^C3^Phanta (circles) or EGFP (triangles) bound to Ni-NTA beads were imaged using frequency domain fluorescence lifetime imaging. Beads were first exposed to cyan photoswitching light (starting at solid arrow) and then photoswitching violet light (starting at dashed arrow). Image data was used to calculate fluorescence lifetimes. An increase in τ_Φ_ for EGFP^C3^Phanta emission on conversion of Phanta to the OFF state indicates a reduction in FRET between EGFP and Phanta. A subsequent decrease in τ_Φ_ on conversion of Phanta to the ON state indicates an increase in FRET. τ_Φ_ for EGFP alone does not alter on exposure to photoswitching light. τ_Φ_ for EGFP alone does not change on exposure to photoswitching light. The average τ_Φ_ across the bead is shown.

The suitability of Phanta as a pcFRET acceptor was further investigated using EGFP^C3^Phanta, a biosensor for caspase 3 activation. The biosensor comprised EGFP fused to the N-terminus of Phanta via a polypeptide linker containing the caspase 3 protease recognition motif (DEVD). The fluorescence emission of EGFP^C3^Phanta bound to Ni-NTA coated beads was monitored by fluorescence microscopy after exposure to photoswitching cyan or violet light. The results show significant and reproducible changes in fluorescence emission between the ON and OFF states of Phanta over at least 20 cycles of photoswitching (Figure 5A).

In a separate experiment, fluorescence lifetime imaging (FLIM) was used to monitor fluorescence emission lifetimes for EGFP^C3^Phanta over a single photoswitching cycle. The average fluorescence phase lifetime (τ_φ_) for EGFP^C3^Phanta bound to a bead increased from 1.5 ns to 2.0 ns (Figure 5B). Subsequent exposure to photoswitching violet light resulted in a drop in τ_φ_ to 1.5 ns, indicating increased FRET. The τ_φ_ (2.5 ns) for a sample of EGFP alone was not significantly affected by exposure to photoswitching light. Collectively, these results indicate that photoswitching of Phanta in EGFP^C3^Phanta between the ON and OFF states results in changes in FRET efficiency.

The thermal stability of the Phanta OFF state was investigated. The fluorescence emission of EGFP^C3^Phanta incubated at different temperatures was monitored over time after Phanta was photoswitched to the OFF state. The results indicate that Phanta thermally relaxes from the OFF to ON state at 21°C and 37°C with a t_1/2_ of 18 min and 5 min, respectively. Since I_off_ is acquired immediately following illumination with photoswitching cyan light, it was estimated that the thermal relaxation of Phanta to the OFF state contributes an error <0.5% in I_off_ intensities under live cell imaging conditions (i.e. 37°C).

**Figure 6:**
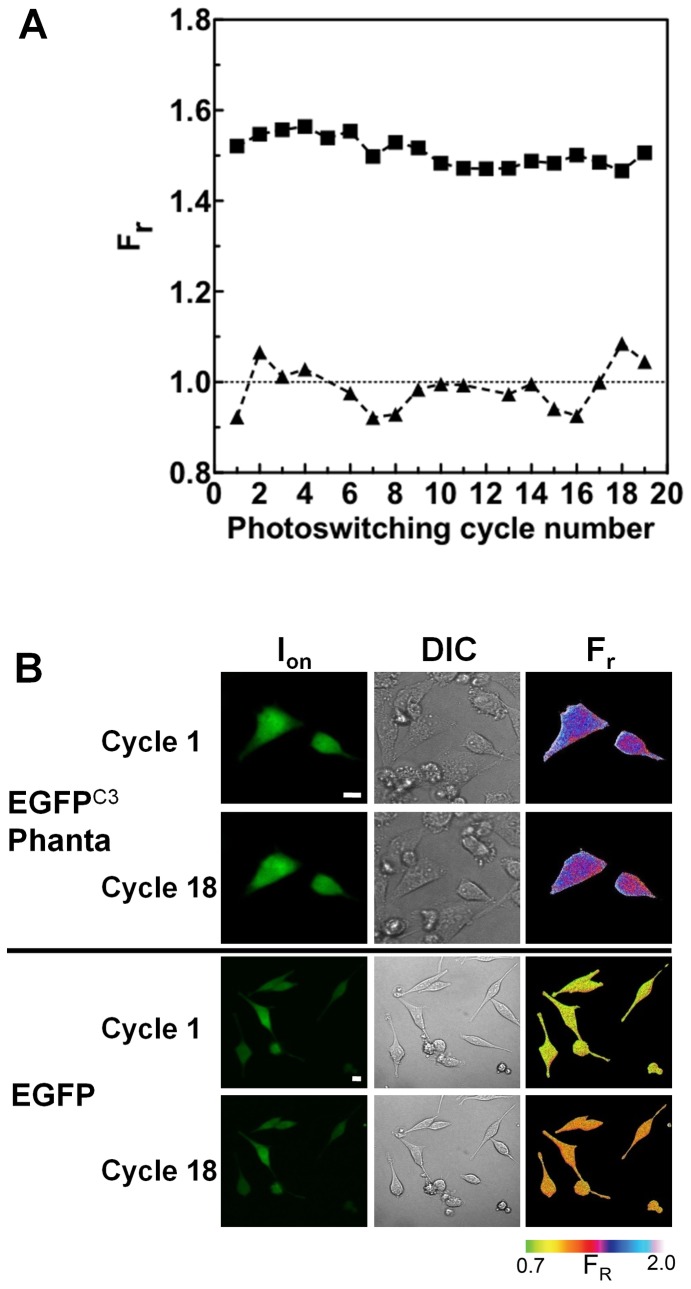
FRET determined in live cells using Phanta is reproducible. (A) Using the approach outlined in Figure 4 live HeLa cells (37°C) within a field of view and expressing EGFP^C3^Phanta or EGFP (donor alone) underwent at 1 min intervals 20 complete cycles of photoswitching. F_r_ was calculated for an individual cell expressing EGFP^C3^Phanta (squares) or EGFP (triangles) after each cycle of photoswitching. (B) Images for cells analysed in A corresponding to I_on_ and DIC acquired after 1 and 18 cycles of photoswitching. The corresponding F_r_ images are shown. Colour bar for F_r_ is shown. Scale bar = 10 µm.

The use of Phanta was investigated in living HeLa cells expressing EGFP^C3^Phanta using confocal scanning fluorescence microscopy. The illumination parameters required for both photoswitching and imaging of EGFP^C3^Phanta were investigated and optimised. The imaging or readout laser (488 nm) power was optimised to avoid unintentional photoswitching of Phanta. In this study the imaging-laser contributed <0.006 to the F_r_ value for any single determination (i.e. single photoswitching cycle). Illumination with photoswitching cyan light was optimised to avoid unintentional photobleaching of the donor. F_r_ was repeatedly determined for a single live HeLa cells expressing EGFP^C3^Phanta or EGFP alone. The results show that donor fluorescence in each of the ON or OFF states, and corresponding F_r_ values were relatively constant over the course of the experiment (Figure 6A and 6B).

**Figure 7:**
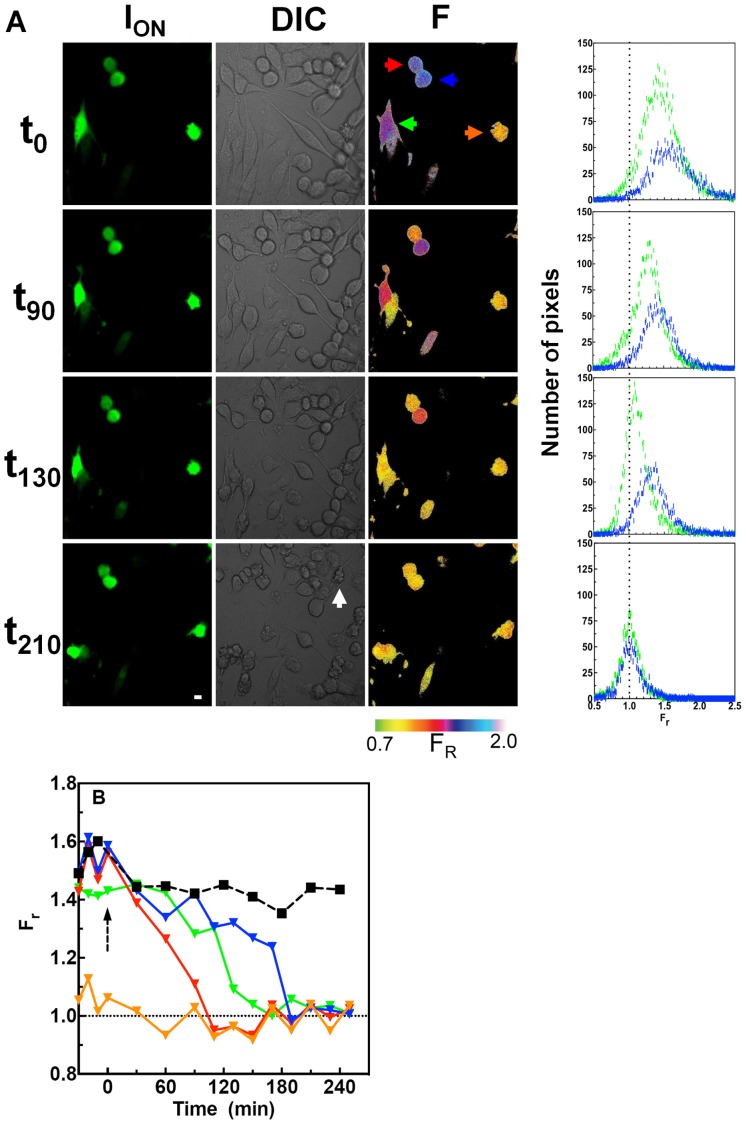
Monitoring activation of caspase 3 in HeLa cells expressing EGFP^C3^Phanta. (A) F_r_ values were determined for HeLa cells expressing EGFP^C3^Phanta and treated with staurosporine. Images were acquired before (t_−30_ to t_0_) and after (t_0_ to t_260_) the addition of STS (5 µM). Time (min) is shown to the left of each panel. Fluorescence emission (I_on_) and F_r_ images are shown for selected time points. The frequency distribution of pixel F_r_ values for two individual cells that change F_r_ with time is highlighted by colour coded arrows (blue and green). The orange arrow shows a cell that does not change F_r_ with time, while the red arrow points to a cell that undergoes rapid reduction in F_r_, indicating apoptosis. Data follows a Gaussian distribution with an R^2^>0.98. The hatched line indicates F_r_ of 1. Apoptosis was induced in non-transfected cells and not expressing EGFP^C3^Phanta (white arrow). Colour bar for F_r_ is shown. Scale bar = 10 µm. (B) F_r_ pixel data for each of the four cells highlighted by arrows in A was Gaussian fitted (R^2^>0.98) and the midpoint of the curve plotted. Each line corresponds in colour to the arrow highlighting an individual cell shown in A. The time point for the addition of STS is indicated (dashed arrow). In a separate control experiment F_r_ values were determined for cells treated with the DMSO vehicle. The F_r_ values for a single control cell not treated with STS are shown (square symbols).

We next investigated whether caspase 3 activation in single living cells could be monitored using EGFP^C3^Phanta and pcFRET. Stauropsorine (STS) is frequently used for the rapid and complete induction of apoptosis [23]. HeLa cells expressing EGFP^C3^Phanta were incubated with STS (5.0 µM), and the resultant changes in FRET efficiency determined at selected times points in single live cells using our protocol (Figure 4). At t_0_ and before the addition of STS, a number of cells in the field of view were observed to be green fluorescent and therefore expressing EGFP^C3^Phanta (Figure 7A). The corresponding FRET image shows that all transfected cells, with the exception of one (orange arrow), have a high F_r_ value (∼1.5) consistent with an intact EGFP^C3^Phanta biosensor, and therefore no significant caspase 3 activity in these cells. The one cell at t_0_ showing evidence of membrane blebbing (orange arrow), a hallmark of apoptotic cells, and an F_r_ approaching unity indicates that this cell contains active caspase 3. At t_90_, a significant drop in the F_r_ is seen for most cells, and by t_130_ all cells show a significant drop in F_r_. The midpoint of the distribution in pixel F_r_ values for each of two cells progressed towards and stopped at unity. The mean F_r_ is shown plotted for each cell over the time course of the experiment (Figure 7B). In a separate experiment control cells not exposed to STS maintain a high F_r_.

## Discussion

Phanta is the first non-fluorescent photochromic member of the GFP-super family. We have shown that Phanta is suitable for use as a pcFRET acceptor in live cells. It has optical properties that are stable over a physiological range of pH, and can be reliably photoswitched between two different absorbing states, each of which show significant differences in spectral overlap with green emitting FPs, a commonly used category of FP. In this study a protocol was developed for use with EGFP as the donor. However, the optical properties of Phanta suggests it is suitable for use as a pcFRET acceptor to other FPs including green emitting FPs excitable with violet light such as T-Sapphire (λ_max_^ex^, 390 nm; R_0_^on^, 55 Å; R_0_^off^, 40 Å) [24] or those with cyan emissions such as Cerulean (λ_max_^em^, 475 nm; R_0_^on^, 54 Å; R_0_^off^, 39 Å [25].

The dark yellow REACh [15] and intense blue Ultramarine [17] are reported to be suitable acceptors for the emission of EGFP. However, use of such dark acceptors to monitor FRET requires donor fluorescence emission lifetimes to be determined using sophisticated and expensive instrumentation. A relatively simple set up including a wide-field fluorescence microscope equipped with a CCD camera or a confocal laser scanning microscope are sufficient to determine FRET using Phanta.

A recent report describes the reversibly photoswitchable rsTagRFP and its suitability for pcFRET applications [12]. rsTagRFP is highly red fluorescent in its ON state (λ_max_^em^, 585 nm; Φ_F_, 0.11) which precludes monitoring of additional probes with similar colour emissions in the same experiment. It is increasingly desirable to monitor multiple events in the same experiment. Since Phanta is non-fluorescent its use will allow inclusion in the same experiment of additional probes whose emissions might otherwise overlap with the acceptor emissions.

The properties of two other categories of dark acceptor suitable for FRET have been published. The chromophore in Ultramarine (λ_max_^abs^, 586 nm; ε, 64,000 M^−1^.cm^−1^) has a *trans* non-coplanar conformation, which is believed to be responsible for its extremely low Φ_F_ (0.001) [17], [26]. REACh2 (λ_max_^abs^, 510 nm; ε, 100,000 M^−1^.cm^−1^, Φ_F_, 0.018) was generated by introduction into EYFP the substitutions Y145W/H148V [15]. The side chains of these amino acids are in close proximity to the tyrosyl moiety of the chromophore, and are thought to promote loss of fluorescence through fast internal conversion. The substitutions required to generate Phanta (Q66M, T73V and H197Q) occupy different positions but are in close proximity to the chromophore, and may therefore promote loss of fluorescence by a similar mechanism suggested for REACh.

The negative photoswitching properties of both Phanta and eCGP123 are similar to those reported for the bright green fluorescent Dronpa [4] whose mechanism of photoswitching involves *cis-trans* isomerisation of the chromophore and changes in key contacts between the chromophore and surrounding protein matrix [27], [28]. It is possible that a similar mechanism is responsible for photoswitching in Phanta. The X-ray crystal structures of eCGP123, Phanta and related variants have been determined [21], and an investigation into the structural basis for the optical properties of Phanta is underway and will be published elsewhere. We anticipate that these data will drive the development of yet other non-fluorescent photochromic proteins for use as acceptors to other fluorescent donors. The red-shifted rsCherryRev [7] or rsTagRFP [12] would make suitable starting points to derive new dark acceptors with red-shifted absorbance spectra and that are complementary to Phanta.

We envisage that Phanta will find use in a range of applications. Phanta will allow increased multiplexing of high-throughput FRET-based assays designed around fluorescence plate readers. Phanta has the potential to improve new super-resolution approaches that rely on FRET [29] by allowing dual channel super-resolution imaging or providing a way to tune fluorescence fluctuation by altering FRET efficiency through photoswitching.

## Materials and Methods

### Mutagenesis

Amino acid positions considered important in determining protein colour were identified by analysing the alignment of amino acid sequences from 15 red and 41 green FPs, together with available crystal structure data. Degenerate oligonucleotide primers targeting selected positions were used to construct a number of different expression libraries based on the bacterial expression vector pET28b. Expression screening of libraries targeting positions 66/67/69/73 and 195/196/197 resulted in bacterial colonies with significantly altered colour and fluorescence properties. Mutants from each library were selected and combined into a new library selected using a PCR assembly approach. Expression of this mixed library resulted in the isolation of Phanta.

### Plasmid construction

Expression vectors encoding EGFP or EGFP^C3^Phanta were constructed as follows. A DNA cassette encoding EGFP flanked by 5′ *Bam*H1 and 3′ nested *Bcl*II/*Not*1 restriction endonuclease sites was cloned into the expression site of pQE9N to produce pQE9N:EGFP. pQE9N was derived from pQE9 by the introduction of a *Not*1 site within the multiple cloning site. A DNA cassette encoding Phanta flanked by a 5′ *Bgl*II site, sequence encoding the caspase 3 protease recognition site (DEVD) flanked by additional amino acids (SDPSGLRSGGDEVDGGSSSRS), and a 3′ *Not*1 site was retrieved by PCR using pET28b:Phanta as template. The PCR product was digested and ligated into the *Bcl*I/*Not*1 sites of pQE9:EGFP to produce the vector pQE9N:EGFP^C3^Phanta and encoding EGFP fused to Phanta via a caspase 3 protease recognition site.

For expression in mammalian cells a *BamH*1/*Not*1 fragment encoding EGFP^C3^Phanta was retrieved from pQE9N:EGFP^C3^Phanta and ligated into the expression site of pCI-neoB to produce pCI-neoB:EGFP^C3^Phanta. pCI-neoB was derived from pCI-neo by eliminating the *BamH*1 site from the backbone of the vector and generating a *BamH*1 site in the multiple cloning site using site directed mutagenesis. A *BamH*1/*Not*1fragment encoding EGFP was retrieved from pQE9N:EGFP and ligated into pCI-neoB to produce pCI-neoB:EGFP.

### Protein expression and purification

Vectors encoding proteins with a C-terminal (pET28b:Phanta) or N-terminal (pQE9N:EGFP and pQE9N:EGFPC^3^Phanta) Hisx6 tag were transformed into *Escherichia coli* (Nova Blue) and proteins expressed and purified as previously described [21]. A single polypeptide with a mobility corresponding to the relative molecular mas of Phanta was observed when purified protein was subjected to SDS-PAGE and gels stained with coomassie blue.

### Photoswitching of isolated proteins

Photoswitching experiments were routinely performed using protein solutions (150 µl; OD_490 nm_ = 1.0) in 20 mM Tris-HCl pH8.0, 300 mM NaCl contained in a single well of a clear 96-well plate. Illumination was performed on a plain white surface using light from a cyan Luxeon Rebel LED (λ_peak_, 505 nm; 130 lm; 0.73 mW/mm^2^) or a 5 mm diameter violet LED (λ_peak_, 405 nm; 60 mcd; 7.8 µW/cm^2^). Temperature was maintained at 37°C. The protein solution was withdrawn after selected periods of illumination points and absorbance spectra were determined using a Cary 50 spectrophotometer (Varian, Australia). Fluorescence excitation and emission spectra were determined using a Cary Eclipse spectrofluorimerter (Varian, Australia). R_0_ values (R_0_^on^ and R_0_^off^) for Phanta paired with selected donor FPs were calculated using the relationship R_0_ = 9.78×10^3^[κ^2^ *n* ^−4^Q_D_J(λ)] [30]. Values of 2/3 were used for the orientation factor (κ^2^) and 1.33 for the refractive index (*n*). Published values were used for individual donor quantum yields (Q_D_). J is the overlap integral between the donor emission and acceptor absorbance, which has been highlighted for both ON and OFF states of Phanta in Figure 2C. R_0_^off^ values were determined for Phanta in its fully OFF state.

Extinction coefficients for proteins were calculated using protein solutions of known protein concentrations and absorbance at λ_max_^abs^. Quantum yields were calculated according to Lakowicz [30] using Rodamine 6G in ethanol as a standard.

### Cell Culture and Fluorescence Microscopy

HeLa cells obtained from the American Type Culture Collection (Manassas, Virginia) were cultured in a 35 mm glass-bottom Fluorodishes (World Precision Instruments) using Dulbecco's modified Eagle's medium (DMEM, High Glucose, HEPES) supplemented with 10% fetal bovine serum (Thermo Scientific) maintained at 37°C and 5% CO_2_. Transfections were performed using FuGENE HD reagent (Roche Applied Science) according to the manufacturer's instructions and cells and visualized 48 h post-transfection. For live cell imaging experiments growth medium was replaced with phenol-free growth medium before imaging. Fluorodishes were maintained at 37°C using a heated stage for the duration of imaging. Apoptosis was induced by the addition of 5 µM staurosporine.

An Olympus IX-81 microscope equipped with an FV-500 confocal laser scanning unit was used to acquire fluorescence image data. Fluorescence images were acquired using a 40× objective. Excitation laser powers (488 nm) were used at intensities optimized so as not to produce significant photoconversion of Phanta during imaging. Intentional reversible photoconversion of Phanta was achieved by short periods of illumination using a filtered mercury arc lamp (100 W) to drive Phanta into the OFF state (460–490 nm; 36 mW/mm^2^) or ON state (395–425 nm; 93 mW/mm^2^), respectively. Illumination times and intensities were optimized to avoid significant donor photobleaching.

Image data for live cells was obtained using the five-step approach outlined in Figure 4 and described as follows. (1). Before the first fluorescence image was acquired the sample was exposed to a brief (0.5 s) flash of violet light (395–425 nm) to ensure that Phanta was fully in its ON state. (2). Fluorescence emission (505–540 nm) due to EGFP was then immediately imaged by laser scanning microscopy on excitation with the readout laser (488 nm) to produce I_on_. (3). The sample was exposed to cyan light to drive Phanta into the ‘OFF’ acceptor state. (4). Fluorescence emission was again imaged to produce I_off_. (5). Image data (I_off_ and I_on_) are used to estimate pcFRET efficiency using the ratio F_r_ = I_off_/I_on_. Cognate pairs of images (I_on_ and I_off_) were first corrected for any relative pixel shift using the public domain software Image-J and the plug-in TurboReg (http://bigwww.epfl.ch/thevenaz/turboreg/). Images were corrected for background and F_r_ calculated the Image-J plug-in Ratio Plus (http://rsbweb.nih.gov/ij/plugins/ratio-plus.html). Look up tables were applied to F_r_ image data. Steps 1–5 were repeated at selected time points.

### Fluorescence lifetime imaging

FLIM was used to determine FRET for EGFP^C3^Phanta bound to beads undergoing reversible photoswitching. FLIM was performed on a frequency domain system supplied by La Vision BioTec (Bielefeld, Germany) coupled to an Olympus IX81 microscope [17]. An acousto-optic modulator was used to modulate continuous wave 488 nm laser light at 80 MHz which was fibre-coupled into the back illumination port of the microscope. Image data was acquired using a Picostar HR full-field detector coupled to the side port of the IX81 consisting of a gated optical intensifier driven by a high rate imager and CCD camera. Two signal generators were implemented in a master/slave configuration to modulate the light source and the Picostar HR with the appropriate phase delays. Illumination was routed through a 403 nm beam splitter and fluorescence emission collected through a 510–550 nm barrier filter. Image data for 10 phase steps over 2π were routinely acquired for the calculation of lifetimes (τ_Φ_ and τ_m_). The system was controlled and image data analysed using ImSpector software (Version 2.3.6; LaVision Biotec). The system was calibrated for fluorescence lifetime using an alkaline solution of fluorescein assuming a fluorescence lifetime of 4.1 ns. Measurements were carried out at 21°C.
