# Correction: Phanta: A Non-Fluorescent Photochromic Acceptor for pcFRET

**DOI:** 10.1371/annotation/b83e925b-2f2a-47b9-b939-0a1eeab18324

**Published:** 2013-10-24

**Authors:** Craig Don Paul, Csaba Kiss, Daouda A. K. Traore, Lan Gong, Matthew C. J. Wilce, Rodney J. Devenish, Andrew Bradbury, Mark Prescott

The affiliation for the third and fourth authors is incorrect. Daouda A. K. Traore and Lan Gong are affiliated not with institution number 2, but with institution number 1, Department of Biochemistry and Molecular Biology, Monash University, Clayton Campus, Melbourne, Victoria, Australia. 

